# The effect of training set on the classification of honey bee gut microbiota using the Naïve Bayesian Classifier

**DOI:** 10.1186/1471-2180-12-221

**Published:** 2012-09-26

**Authors:** Irene LG Newton, Guus Roeselers

**Affiliations:** 1 Department of Biology, 1001 E 3rd Street, Bloomington, IN, 47405, USA; 2 Microbiology & Systems Biology group, TNO, Utrechtseweg, Zeist, The Netherlands

**Keywords:** Honey bee, Gut, Microbiota, Naïve Bayesian classifier, Pyrosequencing, Taxonomy

## Abstract

**Background:**

Microbial ecologists now routinely utilize next-generation sequencing methods to assess microbial diversity in the environment. One tool heavily utilized by many groups is the Naïve Bayesian Classifier developed by the Ribosomal Database Project (RDP-NBC). However, the consistency and confidence of classifications provided by the RDP-NBC is dependent on the training set utilized.

**Results:**

We explored the stability of classification of honey bee gut microbiota sequences by the RDP-NBC utilizing three publically available ribosomal RNA sequence databases as training sets: ARB-SILVA, Greengenes and RDP. We found that the inclusion of previously published, high-quality, full-length sequences from 16S rRNA clone libraries improved the precision in classification of novel bee-associated sequences. Specifically, by including bee-specific 16S rRNA gene sequences a larger fraction of sequences were classified at a higher confidence by the RDP-NBC (based on bootstrap scores).

**Conclusions:**

Results from the analysis of these bee-associated sequences have ramifications for other environments represented by few sequences in the public databases or few bacterial isolates. We conclude that for the exploration of relatively novel habitats, the inclusion of high-quality, full-length 16S rRNA gene sequences allows for a more confident taxonomic classification.

## Background

Microbial ecology studies routinely utilize 454 pyrosequencing of ribosomal RNA gene amplicons in order to determine composition and functionality of environmental communities [1-6]. Where it was once costly to generate libraries of a few hundred 16S rRNA gene sequences, so called next-generation sequencing methods now allow researchers to deeply probe a microbial community at relatively little cost per sequence. Taxonomic classification is a key part of these studies as it allows researchers to correlate relative abundance of particular sequences with taxonomic groupings. These kinds of informative data can also allow for hypothesis generation concerning the community function in the context of a given biological or ecological question. A large number of groups [1-6] utilize the Ribosomal Database Project’s Naïve Bayesian Classifier (RDP-NBC) [7] for the classification of rRNA sequences into the new higher-order taxonomy, such as that proposed in Bergey's Taxonomic Outline of the Prokaryotes [8]. Bayesian classifiers assign the most likely class to a given example described by its feature vector based on applying Bayes' theorem. Developing such classifiers can be greatly simplified by assuming that features are independent given class (naïve independence assumptions). Because independent variables are assumed, only the variances of the variables for each class need to be determined and not the entire covariance matrix. Despite this unrealistic assumption, the resulting classifier is remarkably successful in practice, often competing with much more sophisticated techniques [9,10]. The practical advantages of the RDP-NBC are that classification are straightforward (putting sequences in a predetermined taxonomic context), computationally efficient (building a statistical model based on k-mers in the training set), can analyze thousands of sequences, and does not require full-length 16S sequences (making it an appropriate tool for next generation sequencing based studies). The RDP-NBC relies on an accurate training set – on reference sequences used to train the model and a taxonomic designation file to generate the classification results. Recently the effects of training sets on RDP-NBC performance were investigated [11]; the size and taxonomic breadth of the training set had a significant impact on classification, such that improvements in the confidence of classification of previously “unclassified” sequences were made with a larger, more diverse training set [11].

For environments that lack cultured isolates or are relatively underexplored, researchers are often unable to find an appropriate training set to reveal the taxonomic identity of the extracted sequences [11-13]. However, if previous clone libraries have generated full length, high-quality 16S rRNA gene sequences, then these sequences can be utilized in a training set and taxonomy framework, potentially increasing the precision of the classification provided by the RDP-NBC. Our primary goal in this study was to test the effect of training set on the RDP-NBC-based classification of *Apis mellifera* (European honey bee) gut derived 16S rRNA gene sequences. Insect guts are relatively underexplored and host novel bacterial groups for which there do not exist close, cultured relatives, making taxonomic assignments for 16S sequences and metatranscriptomic data difficult [14-16]. We also sought to improve the classification of sequences from the honey bee gut by the RDP-NBC through the creation of training sets that include full-length sequences identified as core honey bee microbiota as part of a phylogenetic framework first put forward by Cox-Foster *et al.,* 2006 and extended by Martinson *et al*., 2010 [17,18]*.* Below we compare the precision and reproducibility of classification of the honey bee gut microbiota using six different training sets: RDP, Greengenes, arb-silva, and custom, honey bee specific databases generated from each.

## Methods

### Generating a bee-specific seed alignment

Sequences that corresponded to accession numbers published in analyses of bee-associated microbiota and that were near full length (at least 1250 bp) were used to generate the seed alignment for our subsequent analyses (A total of 5,713 sequences were downloaded and 5,158 passed the length threshold) [18-22]. These sequences were clustered at 99% identity, reducing the dataset to 276 representatives. This set of sequences is referred to as the honey bee database (HBDB) throughout and were aligned using the SINA aligner (v 1.2.9, [23]) to the arb-silva SSU database (SSURef_108_SILVA_NR_99_11_10_11_opt_v2.arb) and visually inspected using ARB [24]. We refer to this custom seed alignment as the arb-silva SSU + honey bee alignment (ASHB). To generate a phylogeny we used the ASHB as input to RAxML (GTR + γ with 1,000 bootstrap replicates) using a maximum likelihood framework (Stamatakis 2006). This phylogeny was used to inform the taxonomic designations (see below). In addition, we used the RAxML evolutionary placement algorithm to identify the placement of short reads within this framework (raxmlHPC-SSE3 –f v –m GTRGAMMA –n Placement). Alignment (ASHB) and phylogeny are available in TreeBase at http://purl.org/phylo/treebase/phylows/study/TB2:S13210?x-access-code=52f01c46c780bc323ba5d1d50ea58fd6&format=html).

### Generating a taxonomy file for bee-associated sequences

**Figure 1:**
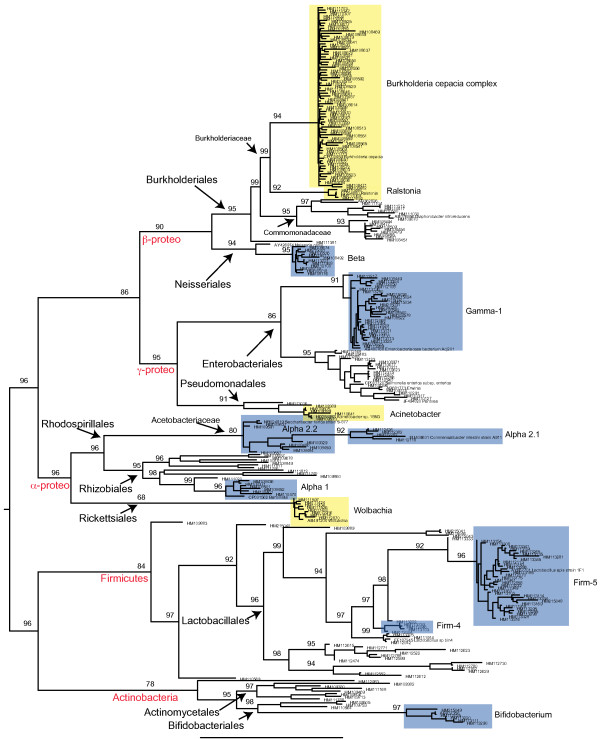
**Phylogenetic relationships for the bacterial species included in the honey bee specific database (with bootstrap support indicated above branches if > 75%).** Class level designations are highlighted in red while lower rank taxonomic designations are indicated using arrows on nodes. Specific clades identified previously in honey bees are colored in blue while novel clades identified here, including cultured isolates and well-described genera (such as *Wolbachia*), are colored in yellow.

Fine scale taxonomic placement (below phylum level) for relatively novel bacterial groups is difficult to accomplish and subject to some debate [13]. In order to taxonomically classify these sequences we utilized the phylogenetic framework generated above (Figure 1) and also queried the RDP (using the RDP-seqmatch tool) for nearest cultured representatives. We used cultured isolates (identified by the RDP-seqmatch tool) to root our phylogeny, generated by the 276 honey bee representative sequences. Based on percent identity to the cultured representative, each sequence in the honey bee dataset was assigned to either the class or the genus level. If the cultured representative was >95% identical to the bee derived sequence, we placed the novel bee sequence in the genus of the cultured representative. If, however, a cultured isolate was not found with identity above 95% for the bee sequence, but they grouped in a clade containing a cultured representative, the bee sequences were placed in the same class and we noted *incertae sedis* in the taxonomy file*.* In addition to this *de novo* generation of taxonomic information for the bee associated sequences, if phylogenetic information (as established by Cox-Foster *et al.,* 2006) was available for any of the Genbank submissions, that information was also included in the taxonomy. For example, names of bee specific groups such as “alpha-2.1” and “beta” (recurring in many bee studies) often appear in the full genbank accession for these sequences. Occasionally the Genbank records list an organism’s full taxonomic designation without considering its placement in the phylogenetic framework previously identified for honey bee guts. For example, *Lactobacillus apis* has a Genbank taxonomy that does not consider it part of the firm-4 group. In our taxonomy, we did not remove the genus and species name but instead consider this sequence to be part of the firm-4 clade at the family level.

### Processing of *pyrosequencing amplicons* from honey bee guts

Raw .sff files corresponding to 16S rRNA gene amplicons from the honey bee gut were downloaded from the DDBJ (DRA000526). The sequences were the result of an amplification of the V1/V2 hypervariable 16S regions with primers 27 F and 338IIR [25]. All data extraction, pre-processing, analysis of operational taxonomic units (OTUs), and classifications were performed using modules implemented in the Mothur software platform [26] as in [25] except where noted below. Information about which colony each sequence came from was retained throughout sequence processing so we could make statistical inferences based on the ecological framework tested previously [25]. Unique sequences were aligned using the “align.seqs” command and the Mothur-compatible Bacterial SILVA SEED database modified to include the ASHB. Out of 70,939 sequences, a total of 4,480 unique, high-quality sequences were retrieved from honey bee guts using this pipeline. Operational taxonomic units (OTUs) were generated using a 97% sequence-identity threshold, as in [25].

### Taxonomic classification and generation of a custom database

To create custom training datasets for Mothur, one requires a reference sequence database and the corresponding taxonomy file for those sequences. We downloaded three pre-existing, Mothur-compatible training sets: 1) the RDP 16S rRNA reference v7 (9,662 sequences), 2) the Greengenes reference (84,414 sequences), and 3) the SILVA bacterial reference (14,956 sequences) each available on the Mothur WIKI page ( http://www.mothur.org/wiki/Main_Page). The datasets are each comprised of both an unaligned sequence file and a taxonomy file. We modified each of these to include the honey bee database (HBDB) to create RDP + bees, GG + bees and SILVA + bees. Using each of these six alternative datasets, we classified the honey bee gut microbiota sequences using the RDP-II Naive Bayesian Classifier [7] and a 60% confidence threshold. In addition, we also tested the ability of the HBDB alone to confidently classify these short reads. Blastn searches were performed using the blast + package (version 2.2.26) using default parameters.

## Results and discussion

### The effect of pre-existing training sets on the classification of honey bee gut sequences

**Figure 2:**
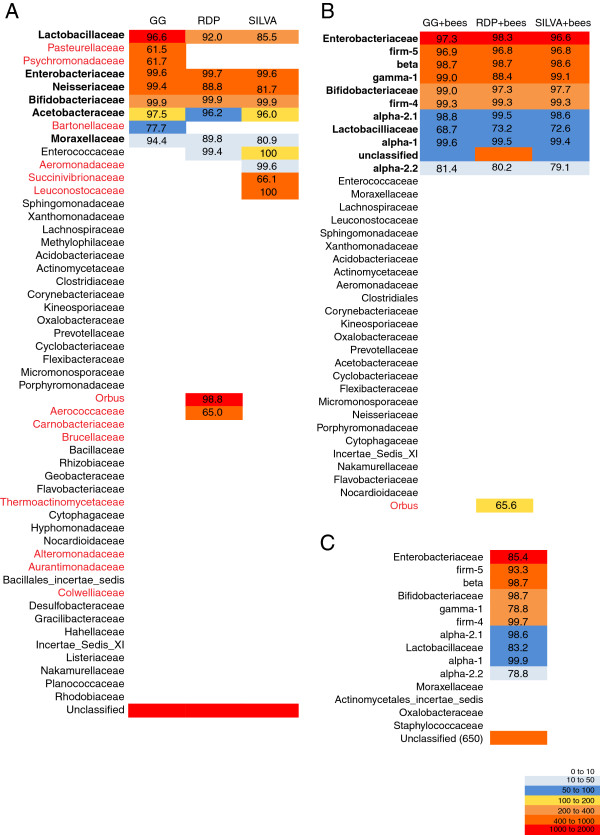
**The effect of training set on the classification of sequences from the honey bee gut visualized by a heat map.** Unique sequences (4,480) were classified using the NBC trained on either RDP, GG, or SILVA (**A**), three custom databases including near full length honey bee-associated sequences RDP + bees, GG + bees, SILVA + bees (**B**), or the near full length honey bee-associated sequences alone (**C**). Family-level taxonomic designations are shown and where taxonomic classifications occur across all three datasets, these are highlighted in bold lettering. Where a classification is unique to one training set, this is highlighted in red font. The average bootstrap score resulting from the classification is provided for each taxonomic assignment.

In order to explore how three heavily utilized pre-existing training sets perform on honey bee gut microbiota, we systematically tested the RDP-NBC in the classification of a 16S rRNA gene pyrosequencing dataset from the honey bee gut. The RDP, Greengenes, and SILVA training sets differ in size, in diversity of sequences, and partly in taxonomic framework. The largest of these datasets, the Greengenes reference, is by far the most diverse, comprised of 84,414 sequences including multiple representatives from each taxonomic class. With regards to taxonomic framework, the RDP relies on Bergey's *Taxonomic Outline of the Prokaryotes* (2nd ed., release 5.0, Springer-Verlag, New York, NY, 2004) as its reference. In contrast, the Greengenes taxonomy assigns reference sequences to individual classifications using phylogenies based on a subset of sequences but also includes NCBI’s explicit rank information [27]. Finally, SILVA, like the RDP, uses Bergey’s Manual of Systematic Bacteriology (volumes 1 through 4), Bergey's Taxonomic Outlines (volume 5), and the List of Prokaryotic names with Standing in Nomenclature [28]. In all three taxonomic references, six taxonomic ranks are predominantly utilized for classification: Domain, Phylum, Class, Order, Family and Genus (although the training set taxonomies differ with regards to the prevalence of suborders, subclasses, etc.). We chose to utilize the SILVA taxonomic nomenclature for the HBDB without observable conflicts across all three training sets for these specific bacterial groups (Figure 2B).

Training set had a significant impact on both the presence and also the predicted abundance of particular taxonomic groups within honey bee guts (Figure 2A). Across all training sets, a total of 10 bacterial classes were predicted to be represented in the bee gut including 27 distinct orders, although certain orders were prevalent only in results from specific datasets, notably Acidobacteriales and Pasteurellales (found predominantly in the Greengenes taxonomic classification) and Bacillales and Aeromonadales (found predominantly in the SILVA results). When comparing classification results at the order level, 3,145/4,480 (70%) of the sequences were classified differently by all three training sets, suggesting a severe inability of the RDP-NBC to place the novel sequences within known cultured isolates and databases. The incongruence between the classifications provided by each training set was magnified as the taxonomic scale progressed from phylum to genus (Table 1). A systematic analysis of congruence between all three training sets for each unique sequence classified revealed that only 595 (~13%) of the sequences concurred in their complete taxonomic classification, down to genus, regardless of training set (Table 1). At the genus level, between the three training sets, RDP and SILVA were the most similar in their classification, agreeing 1017/4480 times. The results provided by the GG based classification were different from those provided by either the SILVA or the RDP datasets, disagreeing ~99% of the time with regards to genus (Figure 2A).

**Table 1:** The taxonomic classification for 16S rRNA gene sequences improves with the addition of custom databases

**Taxonomic Level**	**Congruent Classifications (No. sequences)**	**Incongruent across all three training sets**	**Congruent Classifications with HBDB**
Kingdom	4,480	0	4,480
Phylum	4,465	0	4,478
Class	4,453	4	4,479
Order	2,579	1,335	4,669
Family	1,870	2,784	4,216
Genus	595	2,552	--*

*HBDB sequences were not taxonomically assigned to genus so this level of taxonomic classification was excluded.

The number of 16S rRNA gene sequences from honey bee guts with identical or completely divergent classifications across three widely used training sets (RDP, Greengenes, SILVA) is shown. As the taxonomic levels become more fine, there is an increase in the discordance/errors in taxonomic placement across all three datasets. The addition of honey bee specific sequences greatly improves the congruence across all datasets (last column).

Resultant classification differences could be the product of either 1) differences in the taxonomic framework provided to the RDP-NBC for each sequence or 2) differences in the availability of sequences within different lineages in the training sets used on the RDP-NBC prior to classification. Systematic phylogeny-dependent instability with regards to classification of particular sequences could suggest that representation of related taxonomic groups within the training set is particularly low. To explore the source of classification differences, we investigated the pool of sequences for which training sets altered the classification. In total, 1,335 sequences were unstable in their classification across all three training sets at the order level (Table 1), meaning that they were classified as different orders in each of the three published training sets (RDP, GG, and SILVA). These discrepancies were found to correspond to classifications in three major classes: the α-proteobacteria, γ-proteobacteria and bacilli. Sequences classified as Bartonellaceae by the Greengenes taxonomy were either classified as Brucellaceae (RDP), Rhizobiaceae (RDP), Aurantimonadaceae (SILVA), Hyphomonadaceae (SILVA) or Rhodobiacea (SILVA). Within the γ-proteobacteria, those sequences classified as Orbus by the RDP training set were identified as Pasteurellaceae (GG), Enterobacteriaceae (GG), Psychromonadaceae (GG), Aeromonadaceae (GG and SILVA), Succinivibrionaceae (GG and SILVA), Alteromonadaceae (SILVA), or Colwelliaceae (SILVA). The number of incongruent classifications for sequences identified as Lactobacillaecae by Greengenes were even more astonishing as they were classified as different phyla by use of the RDP or SILVA training sets; these sequences were classified as Aerococcaceae (RDP), Carnobacteriaceae (RDP), Orbus (RDP), Succinivibrionaceae (RDP), Bacillaceae (RDP or SILVA), Leuconostocaceae (SILVA), Listeriacae (SILVA), Thermoactinomycetaceae (SILVA), Enterococcaceae (SILVA), Gracilibacteraceae (SILVA), Planococcaceae (SILVA), Desulfobacteraceae (SILVA).

Training set composition could be affecting the classification results by the RDP-NBC presented above. We explored this possibility by analyzing one particularly striking incongruity between training sets: the classification of sequences as *Orbus.* Only the RDP training set resulted in the classification of honey bee microbiota short reads as *Orbus* and these sequences were used as queries in a blast search against all three training sets (RDP, SILVA, and GG). On average, these *Orbus*-classified sequences were 93% identical to top hits in the RDP training set. They did not find close homologs in the SILVA training set either, the closest top scoring hits being 86% identical (on average). In contrast, in the GG training set, top hits that were 98.6% identical were found and these sequences were classified as γ-proteobacteria, without further taxonomic depth. This result suggests that training set breadth is playing a role in the incongruity observed here. In support of this hypothesis, a large number of short reads were unclassifiable using each training set (1,167 unclassified by SILVA, 1,468 by GG, 2,818 by RDP) and the RDP training set resulted in the least confident classification out of all three with a majority (62%) of the sequences unclassifiable at the 60% threshold. Bootstrap scores resulting from RDP-NBC classifications can be an indicator of sequence novelty [29]; sequences with low scores at particular taxonomic levels may represent new groups with regards to the training set utilized. The average bootstrap scores for each classification at the family level for each of the three training sets was calculated (Figure 2A). Certain sequences were classified with relatively low average bootstrap values, suggesting that these sequences do not have close representatives in the training sets. For example, a low average bootstrap score was observed for the classification of sequences as Succinivibrionaceae by SILVA or as Aerococcaceae by the RDP.

### The use of custom sequences improves the stability of classification of honey bee gut pyrosequences, regardless of training set

In order to improve the classification of honey bee gut derived 16S rRNA gene sequences, a custom database was used to classify unique sequences. The taxonomic classifications in this custom database were generated either by close identity (95%) to a cultured isolate or by the inclusion of cultured isolates in the phylogeny. This phylogeny mirrors those published by others for these bee-associated sequences [18,19,30]; honey bee-specific clades were recovered with bootstrap support >90% (Figure 1). The addition of honey bee specific sequences to each training set not only altered spurious taxonomic assignments for certain classes (notably the δ-proteobacteria are not present in results from these datasets, Figure 2B) but also significantly improved the congruence between classifications provided for each training set (nearly 100% of sequence classification assignments concurred at the family level, Figure 2B). In total, 8 different bacterial orders including Caulobacterales, Rhizobiales, Methylophilales, Neisseriales, Desulfobacterales, Desulfuromonadales, Bacillales, and Pasteurellales were reclassified into the six bee-specific families (Figure 2A,B). Importantly, the large number of unclassifiable short reads observed previously was reduced to <100 sequences when the HBDB was included in the training set (Figure 2B) and the average bootstrap scores for these classifications were generally above 90% (Figure 2B). When we classify these short reads using the HBDB alone (that is, without the inclusion of existing training sets), we see a similar result – the majority of the sequences are classified at a 60% bootstrap threshold (Figure 2C). However, without the additional breadth provided by the GG, SILVA, or RDP training sets, nearly 15% of the short reads (650 out of a total of 4,480) are unclassifiable and average bootstrap scores drop in value, suggesting that the diversity within the bee gut has not been exhaustively characterized by previous 16S rRNA clone library based studies. In contrast to the classifications provided by the published training sets alone (where only 62% of the classifications agreed at the family level across all three training sets), the inclusion of the bee specific sequences dramatically increased the congruence (94% of the sequences agreed at the family level, Table 1). For particular taxonomic orders with high representation (>100 unique sequences) in the honey bee gut, there are particularly few incongruences at the Family level (Figure 2B). Only the RDP + bees training set identifies sequences as *Orbus* classified as either Gamma-1 or Enterobacteriales by the GG + bees or SILVA + bees training sets. It is possible that this error is due to the fact that the RDP training set was the smallest included in this comparative analysis; size and diversity of the training set affects the resulting assignments [11]. We utilized an evolutionary placement algorithm implemented in RAxML to identify the phylogenetic position of short reads classified as *Orbus* by the RDP + bees training set. Indeed, these *Orbus-*like sequences clade within the gamma-1 group (Additional file 1). The spurious placement of these short reads within *Orbus* by RDP was therefore primarily due to the fact that *Orbus* is the closest sequence to gamma-1 found within the RDP training set.

### Biological significance

In the end, the goal of the classifications provided by the RDP-NBC for next generation sequencing datasets is to provide a sense of community structure that may be relevant to function in the environment. There were few incongruities between the HBDB-based taxonomies and those in the existing training sets, primarily because existing training sets did not include sequences identical to these bee-specific groups. Across all three training sets, only 14 sequences were found to be identical to those in the HBDB. The Greengenes training set, for example, included the majority of these identical sequences (12/14) and many closely related sequences (>95% identical across the full length) Additional file 2). However, rarely did our taxonomic designation differ from that in the original training set largely due to the fact that we were looking at the family level, including information about whether or not the sequence had been found in honey bees. As is obvious in Figure 1, certain bee-associated clades include strains identified to the genus and species level (Table 2). Because these strains are bacterial isolates that can be studied with regards to their metabolic capabilities (in some cases, their genome sequences have been completed, see ncbi accession #CP001562), we can begin to determine whether or not there are functional differences relevant in the classification of an organism as either “alpha-2.1” (*Commensalibacter intestini*) or “alpha-2.2” (*Saccharibacter florica*). For example, the pathogen *Bartonella henselae* sequence CP00156 (*B. henselae*) clades with the alpha-1 sequences (Figure 1), a group that often is found in honey bee colonies although the fitness effects on the host are unclear. Additionally, the relevance of the taxonomic designation below the family level for these bee-specific groups remains to be determined.

**Table 2:** Bacterial isolates with genus and species designations that clade within the bee-specific groups

**Bee-specific group**	**Strain taxonomic designation**
Alpha-2.2	*Saccharibacter florica* strain S-877
Alpha-2.1	*Commensalibacter intestini* strain A911
Alpha-1	*Bartonella grahamii* as4aup
Firm-5	*Lactobacillus apis* strain 1 F1

These isolates, and their existing taxonomic information, may inform research into the function of the honey bee gut microbiota.

### Fine scale diversity within the honey bee gut

Using the RDP-NBC and the HBDB custom training sets, a large number of diverse sequences within the honey bee gut were classified in each of the honey bee specific families (Table 3). Although our classification schema does not designate different genera within bee-specific bacterial families, the schema can be used to explore the relevance of fine-scale diversity (at the OTU level) within the honey bee gut (as in [25]). The fine-scale diversity identified previously as present in genetically diverse colonies was found to exist within honey bee-specific bacterial families (Additional file 3), suggesting that host genetic diversity may play a role in shaping the diversity and composition of associated microflora in colonies.

**Table 3:** Diversity of species and unique sequences found within honey bee microbiota

**Family**	**Num. unique sequences**	**OTUs (97% ID)**
Enterobacteriaceae	1621	175
gamma-1	436	48
beta	532	35
Bifidobacteriaceae	363	32
firm-5	929	32
firm-4	253	21
alpha-2.1	90	15
alpha-1	65	13
Lactobacilliaceae	86	12
Flavobacteriaceae	2	2
Leuconostocaceae	2	2
Moraxellaceae	6	2
Sphingomonadaceae	2	2
Xanthomonadaceae	2	2
Actinomycetaceae	1	1
Aeromonadaceae	1	1
alpha-2.2	10	1
Clostridiaceae	2	1
Corynebacteriaceae	1	1
Cytophagaceae	1	1
Enterococcaceae	9	1
Incertae_Sedis_XI	1	1
Kineosporiaceae	1	1
Nakamurellaceae	1	1
Oxalobacteraceae	1	1
Prevotellaceae	1	1

For each family found with honey bee guts (based on SILVA + bees classification) the number of unique sequences and the number of 97% identical operational taxonomic units (OTUs) is shown.

## Conclusions

Insect-associated microbiota can be difficult to classify using existing databases [15]; The lack of cultured isolates or characterized species from insect environments and also the enormous diversity of hosts for the microbial communities is problematic. For example, when predefined, publically available datasets are used to train the RDP-NBC and classify sequences from the honey bee gut, an environment for which there are no cultured representatives, taxonomic classifications are unstable and inconsistent (Figure 2A). In contrast, the HBDB custom training sets effectively and confidently classify the bacteria in the honey bee gut. Results from our classification are consistent with previous studies of the honey bee gut using 16S rRNA clone libraries [17,18], suggesting that the inclusion of environment-specific, high-quality, full-length sequences in the training set can dramatically affect the classification results produced by the RDP-NBC. In addition, the larger, more diverse training sets (SILVA + bees and GG + bees), provided more stable and precise classifications, echoing results of previous studies and suggesting that breadth and depth in the RDP-NBC training set is crucial for more confident taxonomic classifications [11]. This result echoes those of other groups who have found that representation in training sets markedly affects RDP-NBC performance [11,29].

## Abbreviations

RDP-NBC: The Ribosomal Database Project’s Naïve Bayesian Classifier; HBDB: The Honey Bee Database; ASHB: Arb-silva small subunit honey bee alignment.

## Competing interests

The authors declare that they have no competing interest.

## Authors’ contributions

ILGN conceived of the study, implemented the bioinformatics, analyzed resultant data, and drafted the manuscript. GR provided bioinformatics tools, participated in the analysis of the data, and helped to draft the manuscript. All authors read and approved the final manuscript.

## Supplementary Material

Additional file 1 **Table S1.** Total number of operational taxonomic units (97% ID) in either genetically uniform or genetically diverse colonies and classified as one of the honey bee specific taxonomic groups. Click here for file

Additional file 2 **Table S2.** Top scoring blastn hits between full-length, bee specific sequences and the Greengenes training set. Click here for file

Additional file 3 **Figure S1.** Phylogenetic placement of representative short read classified as *Orbus* by the RDP + bees training set. Click here for file
