# Supplementary material for: The effect of training set on the classification of honey bee gut microbiota using the Naïve Bayesian Classifier
Source: BMC Microbiol. 2012 Sep 26;12:221. doi: 10.1186/1471-2180-12-221 (PMC3520854; doi:10.1186/1471-2180-12-221)
Supplement: Additional file 1 — Table S1. Total number of operational taxonomic units (97% ID) in either genetically uniform or genetically diverse colonies and classified as one of the honey bee specific taxonomic groups. [file 1471-2180-12-221-S1.docx]

**Supplementary Table 1.** Total number of operational taxonomic units (97% ID) in either genetically uniform or genetically diverse colonies and classified as one of the honey bee specific taxonomic groups.

| Taxon | Genetically Diverse | Genetically Uniform |
| --- | --- | --- |
| Firm-4 | 44 | 25 |
| Firm-5 | 56 | 46 |
| Alpha-2.1 | 21 | 21 |
| Alpha-2.2 | 4 | 4 |
| Alpha-1 | 16 | 13 |
| Beta | 60 | 38 |
| Gamma-1 | 66 | 51 |
