# Supplementary material for: The effect of training set on the classification of honey bee gut microbiota using the Naïve Bayesian Classifier
Source: BMC Microbiol. 2012 Sep 26;12:221. doi: 10.1186/1471-2180-12-221 (PMC3520854; doi:10.1186/1471-2180-12-221)

**Supplementary Figure 1.** Phylogenetic placement of representative short read classified as *Orbus* by the RDP+bees training set (QUERY below) and the near-full length, bee specific sequences reveals relationship to gamma-1 sequences. Relevant subtree shown.


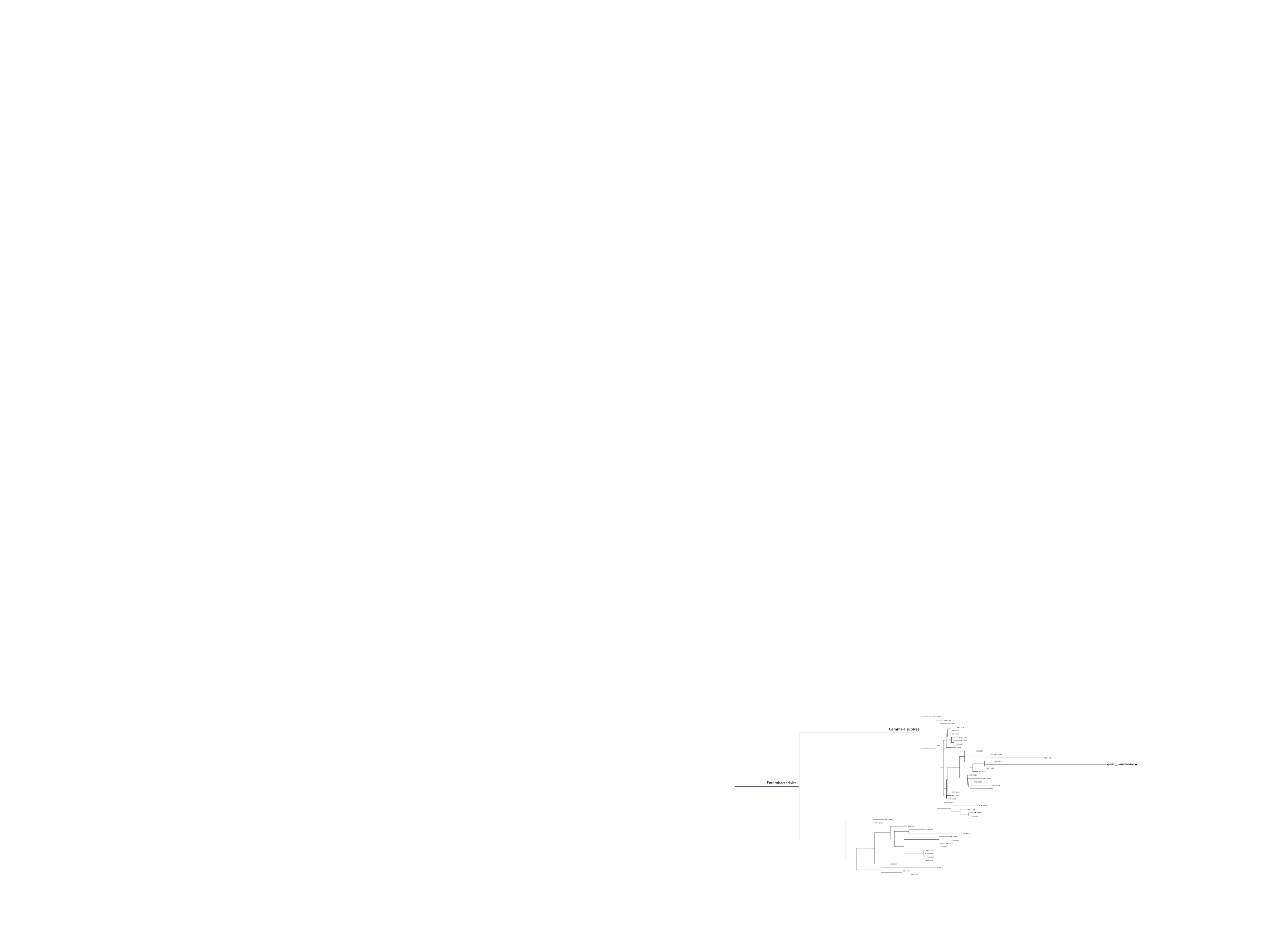

Supplement: Additional file 3 — Figure S1. Phylogenetic placement of representative short read classified as Orbus by the RDP + bees training set. [file 1471-2180-12-221-S3.docx]
